# Urban Microclimates and Their Relationship with Social Isolation: A Review

**DOI:** 10.3390/ijerph22060909

**Published:** 2025-06-06

**Authors:** David B. Olawade, Melissa McLaughlin, Yinka Julianah Adeniji, Gabriel Osasumwen Egbon, Arghavan Rahimi, Stergios Boussios

**Affiliations:** 1 Department of Allied and Public Health, School of Health, Sport and Bioscience, University of East London, London E16 2RD, UK; 2 Department of Research and Innovation, Medway NHS Foundation Trust, Gillingham ME7 5NY, UK; stergiosboussios@gmail.com; 3 Department of Public Health, York St John University, London E14 2BA, UK; 4 School of Health and Care Management, Arden University, Arden House, Middlemarch Park, Coventry CV3 4FJ, UKarahimi@arden.ac.uk (A.R.); 5 Department of Ecotourism and Wildlife Management, Federal University of Technology Akure, Akure 340252, Nigeria; femiyinka2012@gmail.com; 6 Department of Science and Humanities, Leicester College, Leicester LE2 7LW, UK; 7 Department of Industrial Chemistry, Madonna University, Port Harcourt 431125, Nigeria; 8 Faculty of Medicine, Health, and Social Care, Canterbury Christ Church University, Canterbury CT1 1QU, UK; 9 Faculty of Life Sciences & Medicine, School of Cancer & Pharmaceutical Sciences, King’s College London, Strand, London WC2R 2LS, UK; 10 Kent Medway Medical School, University of Kent, Canterbury CT2 7LX, UK; 11 AELIA Organization, 9th Km Thessaloniki—Thermi, 57001 Thessaloniki, Greece; 12 Department of Medical Oncology, Medway NHS Foundation Trust, Gillingham ME7 5NY, UK; 13 Faculty of Medicine, School of Health Sciences, University of Ioannina, 45110 Ioannina, Greece; 14 Department of Medical Oncology, University Hospital of Ioannina, 45500 Ioannina, Greece

**Keywords:** urban heat islands, social isolation, green spaces, microclimates, urban planning, community engagement

## Abstract

Urban microclimates, which include phenomena such as urban heat islands (UHIs) as well as cooler environments created by shaded areas and green spaces, significantly affect social behavior and contribute to varying levels of social isolation in cities. UHIs, driven by heat-absorbing materials like concrete and asphalt, can increase urban temperatures by up to 12 °C, discouraging outdoor activities, especially among vulnerable populations like the elderly and those with chronic health conditions. In contrast, shaded areas and green spaces, where temperatures can be 2–5 °C cooler, encourage outdoor engagement and foster social interaction. This narrative review aims to synthesize current literature on the relationship between urban microclimates and social isolation, focusing on how UHIs and shaded areas influence social engagement. A comprehensive literature review was conducted, selecting sources based on their relevance to the effects of localized climate variations on social behavior, access to green spaces, and the impact of urban design interventions. A total of 142 articles were initially identified, with 103 included in the final review after applying inclusion/exclusion criteria. Key studies from diverse geographical and cultural contexts were analyzed to understand the interplay between environmental conditions and social cohesion. The review found that UHIs exacerbate social isolation by reducing outdoor activities, particularly for vulnerable groups such as the elderly and individuals with chronic health issues. In contrast, shaded areas and green spaces significantly mitigate isolation, with evidence showing that in specific study locations such as urban parks in Copenhagen and Melbourne, such areas increase outdoor social interactions by up to 25%, reduce stress, and enhance community cohesion. Urban planners and policymakers should prioritize integrating shaded areas and green spaces in city designs to mitigate the negative effects of UHIs. These interventions are critical for promoting social resilience, reducing isolation, and fostering connected, climate-adaptive communities. Future research should focus on longitudinal studies and the application of smart technologies such as IoT sensors and urban monitoring systems to track the social benefits of microclimate interventions.

## 1. Introduction

Urban microclimates are localized variations in temperature, humidity, and air quality within urban areas that differ from the broader climate of the surrounding region [1]. These microclimatic differences are primarily shaped by factors such as building density, surface materials like asphalt and concrete, vegetation, and human activities, including transportation and energy usage [2,3,4]. One of the most prominent examples of urban microclimates is the phenomenon of urban heat islands (UHIs), where densely populated urban areas experience significantly higher temperatures than their rural surroundings [3,5,6]. This temperature increase is largely driven by heat-absorbing surfaces and a lack of natural cooling elements such as vegetation [7]. UHIs can result in temperatures that are several degrees higher than in less developed areas, particularly during the night, exacerbating the heat burden on urban residents [7,8].

In contrast, urban green spaces (UGS) and shaded environments created by vegetation, buildings, or specifically designed structures within cities can create cooler microclimates [9,10]. These areas offer refuge from higher temperatures through shade provided by trees, buildings, or other structures, and by leveraging the cooling effect of vegetation through evapotranspiration, the process by which plants release water vapor into the air [11,12]. While UHIs represent a microclimatic phenomenon, shaded areas primarily function as spatial configurations within the urban environment that influence microclimatic conditions. Green spaces and shaded areas mitigate the effects of UHIs and contribute to more comfortable conditions for outdoor activities, fostering environments where social interactions can take place [13].

Social isolation is defined as the objective lack of social contact or interaction with others, which can occur due to physical, environmental, or societal barriers. It differs from loneliness, which is a subjective feeling of being disconnected or unfulfilled in one’s social relationships [14]. Social isolation often involves the absence of meaningful social networks, limited participation in community activities, and reduced interpersonal communication. This state can have profound implications for mental and physical health, increasing the risk of depression, anxiety, cardiovascular diseases, and even premature mortality [15]. In urban environments, factors such as limited access to public spaces, high population density, and environmental conditions like extreme heat can exacerbate social isolation, making it a critical public health and urban planning concern [16]. Social well-being, which can be negatively affected by isolation, is recognized by the World Health Organization as an important aspect of overall health, alongside physical and mental well-being [17,18].

In recent years, social isolation and loneliness have emerged as significant public health concerns, particularly in urban environments [19,20,21]. Despite the high population density of cities, which could theoretically foster social interaction, urban living can contribute to feelings of disconnection and isolation. Research suggests that a third of adults globally experience loneliness, and these numbers are rising due to factors such as rapid urbanization, technological changes, and shifts in social structures [22,23]. Loneliness and isolation are associated with a range of negative health outcomes, including mental health issues like depression and anxiety, as well as physical health conditions such as cardiovascular disease and a heightened risk of early mortality [24].

Public spaces in urban landscapes play a crucial role in facilitating spontaneous social encounters and fostering community connections [25,26,27]. These spaces serve as meeting points where diverse groups of residents can interact, engage in shared activities, and develop social networks that mitigate feelings of isolation. Social interactions, which are crucial for mitigating the effects of loneliness, often take place in outdoor settings—such as parks, squares, or walkable neighborhoods, where people gather and engage with one another [28]. The urban environment, including its microclimatic conditions, plays a key role in facilitating or hindering these interactions. When outdoor temperatures are too high, as is the case in UHIs, people are less likely to spend time in public spaces, which reduces opportunities for spontaneous social encounters. In some cases, extreme microclimate conditions may completely disable rather than merely discourage outdoor activities. This withdrawal from public life can contribute to a deepened sense of isolation and disconnection from the community [29].

Urban microclimates influence social isolation through several interconnected mechanisms. First, thermal discomfort caused by UHIs discourages residents from spending time outdoors, particularly in areas with minimal shading or vegetation. This limits opportunities for casual interactions and participation in community activities, which are vital for fostering social connections [30]. Second, accessibility barriers arise in neighborhoods with poorly designed or inequitable green spaces, leaving vulnerable populations, such as the elderly or low-income individuals, without comfortable places to engage socially [31]. Third, psychological impacts of extreme heat, such as stress or fatigue, can reduce motivation for social engagement, further compounding isolation [32].

Conversely, shaded areas and green spaces mitigate these effects by creating comfortable microclimates that promote prolonged outdoor activity, enabling both structured and spontaneous social interactions [33]. These mechanisms demonstrate how urban microclimates directly affect residents’ ability to participate in communal life, ultimately shaping levels of social isolation. By addressing these pathways, urban planning can create environments that encourage social connectedness and resilience.

For clarity, it is important to distinguish between the various concepts used in this review. Urban microclimates refer to localized climatic conditions within cities that differ from the surrounding region. UHIs are a specific microclimate phenomenon characterized by elevated temperatures in urban areas compared to rural surroundings. Shaded areas and green spaces are physical configurations within the urban landscape that can create cooler microclimates through various mechanisms including evapotranspiration, shade provision, and reduced heat absorption. Social isolation refers to the objective lack of social contacts or interactions, while loneliness refers to subjective feelings of disconnection. This conceptual framework guides our analysis of how physical urban environments influence social behaviors and experiences.

**Figure 1:**
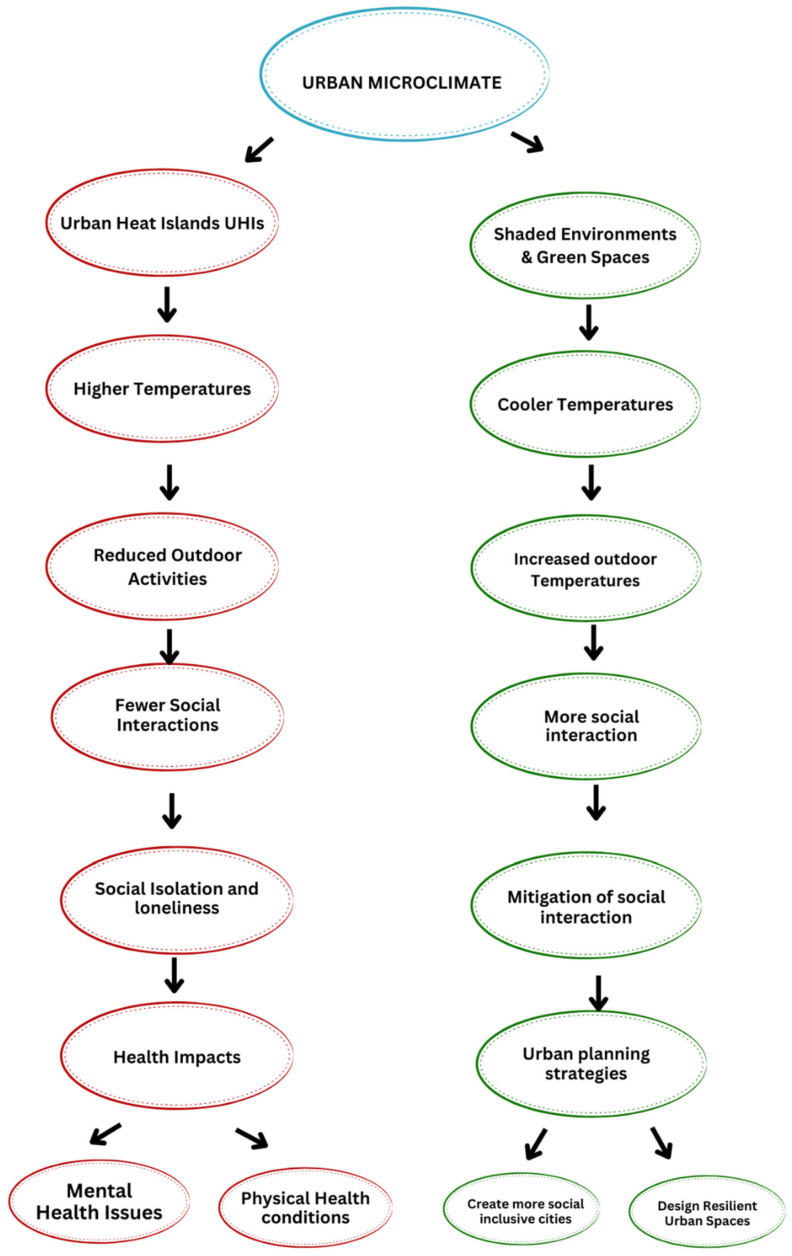
The conceptual framework illustrates the pathways linking urban microclimates to social interaction and health. This figure summarizes the dual influence of urban microclimates. The left pathway shows how urban heat islands (UHIs) elevate temperatures, discourage outdoor activity, and contribute to social isolation and adverse health outcomes; the right pathway demonstrates how shaded environments and green spaces moderate temperatures, promote outdoor engagement, and enhance social connectedness and well-being. The framework emphasizes the central role of urban planning strategies in mitigating negative microclimatic effects and supporting inclusive, resilient communities.

Figure 1 below is a flowchart that highlights the relationship between urban microclimate, social interaction, and health, emphasizing the importance of urban planning strategies in creating more sustainable and equitable cities.

While much attention has been given to social and psychological factors contributing to isolation, there remains a significant gap in understanding how environmental factors, particularly urban microclimates such as UHIs and shaded areas, influence social behavior and community engagement. Despite growing urbanization and the recognized impact of UHIs on public health, research on the direct link between microclimatic variations and social isolation is limited. Few studies have quantified the extent to which these localized environmental conditions shape social interactions, leaving urban planners without clear evidence to guide interventions. Moreover, the role of green spaces and cooling interventions in reducing social isolation remains underexplored, particularly in diverse cultural and geographic contexts.

The specific research question guiding this review is “How do urban microclimates, particularly UHIs and shaded areas, influence social isolation and community engagement, and what interventions can urban planning employ to mitigate these effects?”. The review aims to address this question by synthesizing existing evidence to establish the mechanisms through which microclimates impact social behavior, identifying gaps in current research, and providing actionable recommendations for urban planners. To ensure a cohesive narrative, each section builds on the central theme, beginning with defining key concepts, such as social isolation and urban microclimates, followed by exploring their interconnected effects, analyzing impacts on vulnerable populations, and concluding with urban planning strategies and future research directions. This structure ensures that the review systematically examines the relationship between urban microclimates and social isolation while offering clear insights and practical solutions to bridge existing research and policy gaps.

To clarify the conceptual foundation of this review, Figure 1 presents a framework outlining the interconnected pathways through which urban microclimates, specifically urban heat islands (UHIs) and shaded environments, affect social interaction and health outcomes. This figure serves as an orienting roadmap for the article, illustrating how environmental factors shape social isolation and highlighting the critical role of urban planning interventions in fostering social inclusion and resilience.

## 2. Methods

This narrative review synthesizes current evidence on the relationship between urban microclimates, social isolation, and community engagement. The aim was to explore how environmental factors, particularly UHIs and shaded areas, influence social behavior and to provide actionable insights for urban planning. To ensure a robust and systematic approach, the following methodology was applied.

### 2.1. Literature Search Strategy

A comprehensive search was conducted across multiple academic databases, including PubMed, Web of Science, Scopus, and Google Scholar, to identify relevant peer-reviewed articles and gray literature. The search terms included combinations of keywords such as “urban heat islands”, “microclimates”, “green spaces”, “shaded areas”, “social isolation”, “community engagement”, and “urban planning”. Boolean operators (AND, O, lkk, ojmi R) were used to refine the search, and filters were applied to include studies published in English from 2000 to 2024.

### 2.2. Inclusion and Exclusion Criteria

To ensure relevance, studies were included if they met the following criteria:

1. Examined the relationship between urban microclimates and social behavior;
2. Focused on the effects of UHIs, shaded areas, or green spaces on social isolation or community engagement;
3. Provided empirical evidence, theoretical frameworks, or urban planning interventions addressing microclimatic impacts.

Studies were excluded for the following reasons:

1. Lacked direct relevance to social behavior or urban microclimates;
2. Focused solely on technical climate modeling without behavioral implications;
3. Were opinion pieces or lacked peer-reviewed credibility.

**Figure 2:**
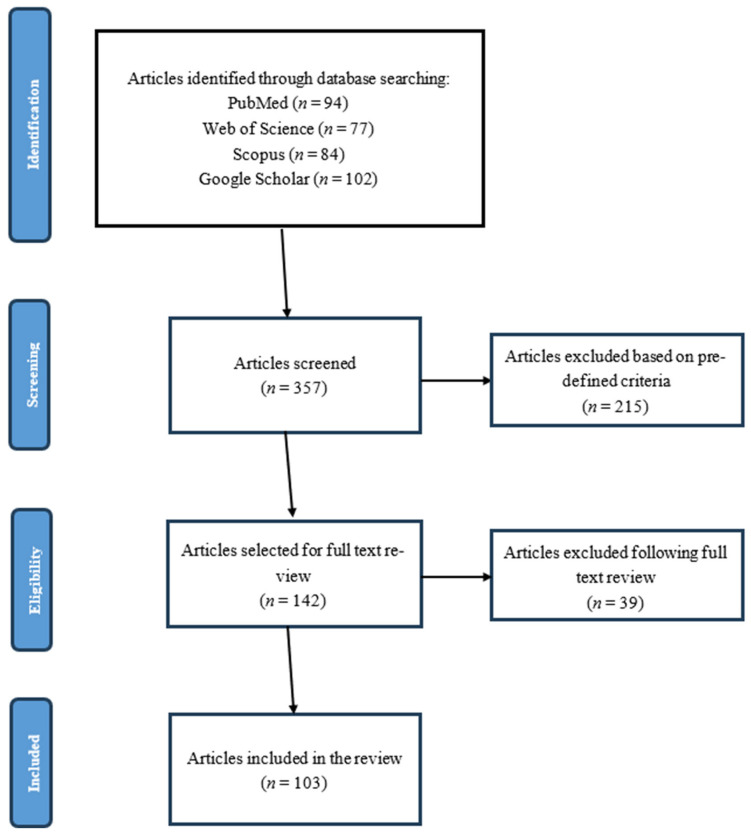
PRISMA flow diagram for article selection process.

The initial search identified 142 potentially relevant articles. After applying the inclusion and exclusion criteria, 103 articles were included in the final review. Figure 2 presents a PRISMA flow diagram documenting the literature selection process.

### 2.3. Data Extraction and Synthesis

The identified studies were reviewed in detail, and key information, such as study objectives, methods, findings, and geographic contexts, was extracted. Priority was given to research with diverse methodologies, including empirical studies, case studies, and theoretical papers, to capture a holistic understanding of the topic. Cross-disciplinary perspectives, such as urban planning, public health, and environmental science, were included to provide a comprehensive synthesis.

Interdisciplinary participation was integral to the data extraction and synthesis process, with researchers from fields including public health, environmental science, urban planning, and social sciences contributing their expertise. This approach ensured a comprehensive analysis that incorporated multiple perspectives on the complex relationship between urban microclimates and social isolation.

### 2.4. Rationale for Source Selection

The sources were selected to address critical gaps in understanding how urban microclimates influence social isolation and to identify actionable strategies for urban planners. A focus was placed on studies with robust methodologies and clear links to both environmental and social outcomes. Geographic and cultural diversity were also considered to ensure applicability across various urban contexts.

## 3. The Concept of Urban Microclimates

Urban microclimates refer to localized environmental conditions within cities that differ significantly from the broader regional climate [34]. These variations are primarily driven by human-made alterations to the natural landscape, such as the replacement of vegetation with built structures and the extensive use of materials like asphalt and concrete, which have distinct thermal properties [35,36]. The microclimates created by these urban modifications can significantly influence local weather conditions, air quality, and temperature fluctuations, particularly in densely populated cities. Two of the most notable features of urban microclimates are UHIs and the cooling effect provided by shaded areas and green spaces [36,37]. Each of these phenomena has distinct implications for the well-being of city residents and their patterns of social interaction, especially as urbanization accelerates globally.

### 3.1. UHIs

UHIs occur when natural surfaces, such as soil, grass, and trees, are replaced by heat-absorbing materials like asphalt, concrete, and buildings. These materials not only absorb solar radiation but also prevent the dissipation of heat, resulting in localized warming, particularly during the night. Research shows that UHIs can cause urban temperatures to be 1–3 °C higher than in surrounding rural areas, with extreme cases reporting temperature differences of up to 12 °C [38,39,40,41]. This increase in temperature is further compounded by factors like reduced vegetation, increased air conditioning use, and waste heat from vehicles and industrial activities.

The health and social consequences of UHIs are substantial, particularly during heatwaves, when the risk of heat-related illnesses such as heat exhaustion and heatstroke increases [42]. Vulnerable populations, including the elderly, children, individuals with chronic illnesses, and those without access to air conditioning, are disproportionately affected [43,44]. These groups are more likely to find outdoor activities physically uncomfortable or unsafe during periods of extreme heat, contributing to their social isolation. Studies have shown that in UHI-affected areas, people are less likely to engage in spontaneous social interactions, as they avoid outdoor spaces that might otherwise serve as venues for community engagement [45,46]. Furthermore, the psychological toll of staying indoors during prolonged heat events can exacerbate feelings of loneliness, as people become less inclined to participate in social or recreational activities.

It is worth noting that while UHIs generally have negative impacts during summer months, they can provide thermal benefits during winter, creating warmer microclimates that may encourage outdoor activity when temperatures would otherwise be prohibitively cold [47,48]. This seasonal variation in the effects of UHIs on social behavior highlights the complexity of urban microclimatic influences on community engagement.

### 3.2. Shaded Areas and Green Spaces

In contrast to the oppressive heat generated by UHIs, shaded areas and green spaces provide cooler microclimates that offer significant relief from extreme temperatures. Vegetation and trees play a key role in these cooler environments by shading surfaces from direct sunlight and reducing the overall temperature through evapotranspiration, where water is absorbed and released as vapor, cooling the surrounding air. Research has demonstrated that urban areas with significant tree cover can be up to 5 °C cooler than nearby UHI zones, providing a more comfortable environment for residents [7,49,50]. Rather than simply lowering temperatures during warm seasons, green spaces provide thermal regulation year-round, including an insulation effect during colder months that can retard heat loss from urban surfaces [51,52]. This thermal regulation effect makes these spaces valuable for promoting outdoor activity throughout the year.

Shaded areas and green spaces are not only important for temperature regulation but also serve as critical social hubs within cities [53]. Parks, tree-lined streets, and green corridors offer inviting spaces for residents to gather, engage in physical activities, or simply relax. These spaces are particularly important in fostering social interactions and community cohesion, as they encourage people to spend time outdoors and interact with others. A growing body of evidence suggests that access to green spaces can reduce feelings of social isolation, improve mental health, and enhance residents’ overall sense of well-being [54]. Moreover, the presence of these spaces has been linked to increased social cohesion, with people living near well-maintained green areas reporting higher levels of neighborhood satisfaction and trust among residents.

In addition to the mental and social benefits, green spaces also contribute to physical health by encouraging outdoor physical activity, which can mitigate some of the adverse health effects associated with sedentary lifestyles and social isolation [55]. The accessibility and design of these spaces play a crucial role in determining their effectiveness in promoting social interactions. For example, shaded walkways, open plazas with tree cover, and community parks designed with seating and recreational areas provide ideal settings for both structured and spontaneous social gatherings. These elements create opportunities for people to come together, reducing the barriers to social engagement and fostering a sense of community within the urban environment.

## 4. The Relationship Between Urban Microclimates and Social Behavior

Urban microclimates can have a profound impact on social behavior, particularly in terms of how outdoor urban spaces are utilized for social interaction [56,57]. The interplay between localized temperature variations and social engagement can either encourage or deter community participation in public life [58]. Social behavior in urban settings is highly dependent on environmental comfort, and when outdoor spaces become inhospitable due to extreme heat or cold, social interactions are likely to diminish [59]. Table 1 provides a structured overview of how urban microclimates, including UHIs, shaded areas, seasonal variations, and time of day, impact social behavior, public space usage, mental and physical health, and social isolation.

## 5. Outdoor Temperature and Social Interaction

One of the most direct ways urban microclimates influence social behavior is through their impact on outdoor temperature. In cities affected by UHIs, excessive heat can significantly reduce the likelihood of outdoor activities. As urban areas absorb and retain heat, particularly during hot weather, outdoor spaces such as parks, squares, and streets may become uncomfortable or even completely uninhabitable, particularly during heatwaves. Studies have consistently shown that high temperatures are associated with reduced pedestrian activity, as people avoid public spaces in favor of indoor environments with air conditioning [88,89]. The result is fewer opportunities for spontaneous social interactions, which can contribute to feelings of isolation, particularly for individuals who rely on outdoor spaces for social engagement.

For example, a study found that during periods of extreme heat, the use of outdoor public spaces decreased by up to 30%, with parks and open-air markets seeing the most significant drops in visitor numbers [90]. The reduction in outdoor social activities is more pronounced among vulnerable populations such as the elderly, children, and those with underlying health conditions, who are at greater risk of heat-related illnesses [91]. In these cases, the fear of heat exposure further reduces the likelihood of venturing outside, compounding the problem of social isolation.

In contrast, cooler microclimates—often found in shaded or vegetated areas—encourage outdoor activity and social interaction. Shaded parks, tree-lined streets, and areas with artificial shading systems create comfortable environments where residents can spend extended periods outdoors. These areas promote social behavior by providing a more inviting atmosphere for both structured social gatherings and spontaneous interactions [53]. The cooling effect of shaded spaces has been linked to improved mental well-being, as individuals are more likely to engage in physical activity, relax outdoors, and form connections with others in their community [92,93]. Additionally, the esthetic and psychological benefits of green spaces have been shown to enhance people’s sense of belonging, reduce stress, and foster a sense of community.

Seasonal variations also play a critical role in how urban microclimates affect social behavior. In summer, when temperatures peak, UHIs often exacerbate heat-related discomfort, making outdoor spaces unappealing for social interaction [94,95]. However, in colder months, UHIs may have the opposite effect, providing a warmer environment for outdoor activities compared to rural areas or suburban zones that lack the same thermal retention [96,97]. In winter, urban areas with UHIs may experience milder temperatures that make outdoor spaces more attractive for social engagement, particularly in regions where cold weather dominates much of the year [65]. Warmer urban microclimates can thus serve as a mitigating factor against social isolation in winter, encouraging outdoor gatherings and community events that would otherwise be limited by low temperatures.

Time of day also influences how urban microclimates shape social behavior. In areas affected by UHIs, the most comfortable periods for outdoor social interaction tend to be early morning and late evening, when temperatures are cooler [98]. During the hottest part of the day, typically in the afternoon, public spaces are often deserted, as people seek refuge indoors. However, shaded areas and green spaces can extend the usability of outdoor spaces throughout the day by providing cooler microclimates that counterbalance the midday heat. This diurnal fluctuation in comfort levels has been observed in numerous studies, which highlight that outdoor activities, such as walking, exercising, or socializing in parks, tend to peak in the morning and evening hours in UHI-affected cities [96,99]. Conversely, in colder months or regions, the heat retention of urban surfaces can make evening activities more comfortable than in rural or suburban areas, where temperatures drop more quickly after sunset [46]. This creates opportunities for social interactions during colder seasons, as urban residents are more likely to use public spaces for evening gatherings and events when the temperature is more moderate.

## 6. Social Isolation and Urban Microclimates: A Causal Pathway

Urban microclimates play a pivotal role in shaping social behavior and influencing social isolation, especially in densely populated areas where environmental conditions can significantly impact daily life [75]. The connection between microclimates, such as UHIs and shaded areas, and social isolation can be understood through a causal pathway. This pathway explores how the built environment, particularly temperature variations, affects residents’ social interactions and their sense of belonging to the community [76,77]. The environmental discomfort created by UHIs often results in social avoidance, while shaded areas and green spaces act as vital social hubs that encourage community engagement and reduce isolation.

Social isolation refers to the objective lack of social contacts, while social interaction encompasses various forms of engagement between individuals, from casual conversations to organized group activities. Spontaneous social interactions—unplanned encounters that occur in public spaces—are particularly vulnerable to microclimate conditions. The environmental discomfort created by UHIs often results in social avoidance, while shaded areas and green spaces act as vital social hubs that encourage community engagement and reduce isolation.

### 6.1. Urban Heat and Social Avoidance

Prolonged exposure to extreme heat, a defining feature of UHIs, has been shown to discourage individuals from spending time outdoors, leading to a phenomenon of social avoidance [100]. When temperatures rise significantly, particularly in areas with limited green spaces or shading, outdoor environments become inhospitable [101,102]. As a result, residents retreat indoors to seek cooler conditions, avoiding parks, sidewalks, plazas, and other public spaces that would typically facilitate casual social interactions. Studies show that high temperatures reduce the likelihood of people engaging in outdoor activities such as walking, exercising, or meeting friends in communal spaces, directly limiting their opportunities for spontaneous social encounters [33,103,104].

The behavioral pattern of staying indoors to escape the heat can have a cumulative effect, gradually reinforcing social isolation. As residents become accustomed to avoiding outdoor spaces during hot periods, their interactions with neighbors and community members diminish [105]. Over time, this avoidance leads to reduced social connectedness and an increased sense of loneliness. For vulnerable populations, such as the elderly or those with preexisting health conditions, the risk of social isolation is even higher, as they are more sensitive to heat and more likely to withdraw from outdoor activities [106]. This withdrawal from public life further exacerbates the mental health risks associated with loneliness, including depression, anxiety, and stress, creating a vicious cycle of isolation [107]. Furthermore, urban environments characterized by UHIs may lack the infrastructure needed to mitigate the negative effects of extreme heat [108]. The absence of cooling stations, shaded walkways, or sufficient green spaces limits residents’ ability to engage with the public realm, reinforcing patterns of avoidance. Over time, residents in these areas may experience a sense of disconnection from their communities, contributing to a decline in social cohesion and an increased risk of mental health issues tied to isolation.

### 6.2. Shaded Areas as Social Hubs

In contrast to the isolating effects of UHIs, shaded areas and green spaces serve as essential social hubs within urban environments [8,29]. These cooler microclimates provide a refuge from the heat, making outdoor spaces more comfortable and accessible for residents. Shaded areas, whether created by trees, canopies, or strategically designed buildings, invite people to spend more time outdoors, promoting a sense of community and social cohesion. Green spaces, in particular, have been shown to foster social interactions by providing inviting, open areas for recreational activities, relaxation, and gatherings [109].

The presence of these shaded environments supports both structured and spontaneous social activities. Parks, for example, often serve as venues for organized events such as community festivals, sports, or group exercise classes [110,111]. They also act as informal meeting points, where residents can engage in casual conversations, connect with neighbors, or simply enjoy the natural environment in proximity to others [112]. The psychological and physical comfort offered by shaded areas encourages longer stays and repeated visits, increasing the likelihood of social encounters and the development of stronger community ties.

To better understand social interactions in these spaces, it is important to define them clearly; “spontaneous social interactions” refer to unplanned encounters between individuals that occur naturally in public spaces, while more formal or structured interactions include planned community events and organized activities. Numerous studies have highlighted the relationship between access to green spaces and improved social cohesion [33,53,113]. In neighborhoods with ample tree cover and well-maintained parks, residents report feeling more satisfied with their communities and more connected to those around them. The cooling effects of these spaces, combined with their esthetic and recreational value, enhance the overall well-being of residents, reducing stress and promoting a positive sense of belonging. By providing comfortable settings for social interaction, shaded areas help mitigate the risk of social isolation, particularly in urban environments, where residents may otherwise be disconnected due to the pressures of modern city life.

Moreover, shaded areas can act as inclusive spaces, drawing in a diverse cross-section of the population. Unlike UHIs, which often disproportionately affect vulnerable groups, shaded spaces offer relief and comfort to all residents, regardless of age, physical ability, or socioeconomic status [114]. This inclusivity strengthens the role of these areas as social hubs, where people from different backgrounds can interact, fostering a sense of unity and shared identity within the community.

It is important to note that the demographic profile of users of shaded spaces can vary significantly. Research suggests that factors such as age, gender, cultural background, and socioeconomic status influence how different populations utilize and benefit from these spaces. Understanding these patterns is crucial for designing inclusive green spaces that serve the needs of diverse urban populations.

## 7. Impact on Vulnerable Populations

Elderly individuals, people struggling with mental health, children, and those with chronic health conditions are grouped under “vulnerable populations”, due to their heightened sensitivity to the adverse effects of urban microclimates [115]. These groups are disproportionately impacted by UHIs because of physical, developmental, or health-related limitations that reduce their ability to adapt to extreme heat. For instance, the elderly often experience reduced thermoregulation and mobility issues, while children are more susceptible to heat stress due to their developing physiology [116]. Similarly, individuals with chronic health conditions or mental health challenges may face increased barriers to accessing public spaces, exacerbating their risk of social isolation. Cultural norms may also affect vulnerability; in some cultures, people are accustomed to wearing more clothing despite heat, potentially increasing their vulnerability to heat stress.

Addressing the specific needs of these populations highlights the importance of equitable urban planning and targeted interventions to create inclusive, climate-resilient spaces. Table 2 highlights how UHIs and the lack of shaded spaces disproportionately affect vulnerable populations, while shaded areas and green spaces offer significant benefits for reducing social isolation, improving mental health, and encouraging physical activity.

## 8. UHIs and Their Effect on Vulnerable Populations

UHIs create a hotter microclimate in densely built areas, resulting from the absorption of heat by materials such as asphalt, concrete, and buildings, which retain heat longer than natural landscapes [131]. The effects of this heat are felt most acutely by vulnerable populations, particularly the elderly and those with chronic health conditions. As people age, their bodies become less efficient at regulating temperature, making older adults more susceptible to heat-related illnesses, such as heat exhaustion, heat stroke, and dehydration [132]. Chronic conditions such as cardiovascular disease, diabetes, and respiratory disorders also heighten vulnerability to extreme temperatures, which can exacerbate these underlying health issues [133].

For many elderly individuals living in urban areas affected by UHIs, high temperatures act as a significant deterrent to going outdoors. Research shows that older adults are more likely to stay indoors during periods of extreme heat, limiting their access to public spaces where they might otherwise engage in social activities [106,134]. The avoidance of outdoor spaces due to heat discomfort leads to reduced physical activity, which is crucial for maintaining health and preventing further isolation. Additionally, heat-related discomfort can discourage participation in community events, outdoor exercise, and casual interactions with neighbors, all of which are essential for preventing loneliness and fostering social connections.

Studies indicate that elderly residents living in UHI-prone areas report higher levels of social isolation and mental health issues, such as depression and anxiety [19]. The psychological stress of extreme heat, combined with physical health vulnerabilities, compounds the social isolation experienced by many older adults. Moreover, low-income elderly individuals are disproportionately affected, as they are more likely to live in areas with fewer green spaces, poorer access to air conditioning, and less infrastructural investment to mitigate heat exposure [135]. For these populations, UHIs not only increase the physical burden of heat but also reduce opportunities for meaningful social engagement, which is critical for emotional and psychological well-being.

### 8.1. The Impact on Children and Individuals with Chronic Health Conditions

Children are another population that a particularly vulnerable to the effects of UHIs [136,137]. Young children are less capable of regulating their body temperature than adults, making them more susceptible to heat stress. High temperatures can limit children’s opportunities for outdoor play and physical activity, which are crucial for their physical and social development [138]. Outdoor spaces like parks and playgrounds are essential venues where children interact with peers, develop social skills, and engage in exercise. However, when these spaces become too hot due to UHIs, children are often kept indoors, limiting their ability to engage in these critical developmental activities.

Moreover, children living in low-income, high-density urban neighborhoods often have less access to green spaces and shaded areas where they can safely play during hot weather [139]. This disparity in access exacerbates health and social inequalities, as children from disadvantaged backgrounds are more likely to experience both physical inactivity and social isolation during hot periods. The long-term impact of reduced outdoor play due to heat can affect children’s overall development, including their cognitive, social, and emotional well-being [140]. For individuals with chronic health conditions, such as cardiovascular disease, respiratory problems, and obesity, the effects of UHIs are particularly harmful. Extreme heat can exacerbate these conditions, making it more difficult for affected individuals to engage in physical activity or even leave their homes. Physical inactivity, combined with isolation, creates a cycle that further deteriorates health outcomes for people with chronic conditions [141]. The lack of access to cool, comfortable outdoor environments forces these individuals into prolonged periods of isolation, increasing their risk of depression, anxiety, and other mental health issues.

### 8.2. Shaded Areas and Green Spaces as a Solution for Vulnerable Populations

In contrast to the isolating effects of UHIs, the availability of shaded areas and green spaces can serve as a critical solution for vulnerable populations, providing safe and comfortable environments for outdoor activities and social engagement [142]. Green spaces, such as parks, tree-lined streets, and community gardens, offer cooler microclimates that can reduce the physical burden of heat and encourage people to spend more time outdoors. These areas promote physical activity, social interaction, and mental well-being, all of which are essential for mitigating the harmful effects of social isolation.

For older adults, access to well-maintained, shaded public spaces can increase social participation and provide opportunities for physical activity, such as walking, tai chi, or simply relaxing outdoors with others. Studies have shown that older adults who have regular access to green spaces report lower levels of loneliness and higher levels of life satisfaction [143,144,145]. Shaded areas also serve as informal gathering spots where older adults can meet with friends or engage in community activities, helping to foster a sense of belonging and social cohesion.

For children, shaded playgrounds and parks provide safe environments for play and physical activity, even during hot weather. Access to these spaces supports children’s social development by enabling them to interact with peers, develop friendships, and engage in group activities that promote teamwork and communication. Research suggests that children who have regular access to green spaces exhibit better mental health outcomes, improved attention spans, and enhanced cognitive development [146,147,148].

Similarly, for individuals with chronic health conditions, green spaces provide a refuge from the heat and a setting for low-impact physical activity, which can help improve their health outcomes. The opportunity to exercise or simply enjoy a cooler outdoor environment can reduce stress and improve overall well-being, while social interactions in these spaces help combat feelings of isolation. Accessible green spaces also provide a sense of agency and control for individuals with chronic health conditions, offering them a safe space to engage in physical and social activities without the risk of heat-related health complications [149].

While access to green spaces provides numerous benefits, it is important to consider the factors that may hinder users from utilizing these spaces effectively. Both user-related factors (such as mobility limitations, time constraints, and health conditions) and environment-related factors (such as safety concerns, maintenance issues, and inadequate shade) can limit the accessibility and usability of green spaces. Addressing these barriers is essential for maximizing the social and health benefits of urban green areas for vulnerable populations.

## 9. Urban Planning and Policy Implications

Urban planning and policymaking are crucial in addressing the challenges posed by urban microclimates, particularly UHIs, and ensuring that cities remain socially inclusive, healthy, and resilient [150,151,152]. As cities continue to expand and face the increasing threats of climate change, it is imperative that urban planners integrate strategies that mitigate the negative effects of microclimates on social behavior and well-being. These strategies must consider the creation of more livable, accessible, and socially engaging environments that can counteract the adverse effects of UHIs while fostering social cohesion and community resilience.

One of the most effective ways to mitigate the impacts of UHIs is through urban greening initiatives. Increasing vegetation, particularly in densely populated urban areas, can significantly reduce localized temperatures by providing shade and cooling through the process of evapotranspiration [153]. Trees, shrubs, and green spaces help absorb less heat compared to concrete and asphalt, creating cooler microclimates that encourage outdoor activities and social interactions [154]. Green roofs and walls are also increasingly being recognized as valuable tools in reducing urban heat. By adding vegetation to rooftops and building facades, cities can not only reduce temperatures but also improve air quality, increase biodiversity, and enhance the esthetic appeal of urban environments [155].

In addition to greening initiatives, urban design plays a critical role in mitigating the effects of UHIs. Optimizing building materials and urban layouts can help reduce heat absorption and retention. For example, the use of reflective and light-colored materials for pavements and buildings can minimize heat buildup, while strategic placement of buildings can maximize natural shading and airflow [156]. Urban designs that prioritize pedestrian-friendly areas, shaded walkways, and open plazas can make outdoor spaces more comfortable, encouraging social activities and reducing the isolation that excessive heat can foster [157]. Furthermore, the implementation of cooling infrastructure such as water features, fountains, and urban ponds can also help reduce ambient temperatures, offering relief to residents and providing inviting spaces for social gatherings [158]. Integrating these elements into urban design not only improves the microclimate but also enhances the quality of public spaces, making them more attractive for social use.

While greening efforts can mitigate heat, creating shaded areas and accessible green spaces is critical for promoting social interaction and reducing isolation, especially for vulnerable populations [159]. Urban planners should prioritize the development of parks, shaded walkways, and tree-lined streets that provide comfortable environments for outdoor activities. Such spaces can serve as essential social hubs where people can meet, engage in recreational activities, and form connections with their communities. It is important to note the distinction between greenery coverage and accessibility. While increasing the total amount of green space in a city is important, ensuring equitable access to these spaces is equally critical. Urban planning strategies must balance both considerations, as evidence shows that accessible but smaller green spaces can have greater social benefits than larger, less accessible ones.

Ensuring that green spaces are inclusive and accessible is essential to fostering social equity. Policymakers must consider the distribution of green spaces across cities, ensuring that all neighborhoods—especially low-income and marginalized communities—have access to these vital resources. Research shows that access to green spaces is often uneven, with disadvantaged communities experiencing greater exposure to UHIs and fewer opportunities for social engagement [31,135]. By prioritizing the equitable distribution of green spaces, cities can address these disparities, improving both physical and social health outcomes for vulnerable populations.

The design of green spaces should also consider the diverse needs of urban residents, including children, the elderly, and individuals with disabilities [31,160]. Parks equipped with shaded play areas, accessible walking paths, seating areas, and community event spaces can accommodate a wide range of social and recreational activities, encouraging greater use and interaction. For example, shaded seating areas in parks provide elderly residents with comfortable places to gather and socialize, while shaded playgrounds allow children to play safely, even in hot weather [161].

### Policy Interventions for Social Resilience

Urban planners and policymakers must also implement programs that focus on increasing social resilience, particularly in response to climate-induced challenges like heatwaves. These interventions can include the development of cooling centers, which provide air-conditioned public spaces where residents, particularly vulnerable groups, can find relief during extreme heat events. While cooling centers are primarily indoor environments, they can be designed to foster social interaction and community building, complementing the role of outdoor green spaces in promoting social connection. Cooling centers can double as community hubs where residents can socialize and access critical resources, reducing the isolation often experienced during prolonged periods of heat [162,163].

The promotion of urban park programming is another valuable policy intervention [111]. By hosting community events, festivals, exercise classes, and cultural activities in parks and green spaces, cities can encourage residents to utilize these spaces and engage with one another. Such programming not only promotes social cohesion but also enhances residents’ mental and physical well-being by providing regular opportunities for outdoor social engagement.

There are three specific approaches to policy interventions that merit particular attention:

1. Development of cooling centers and shaded public spaces;
2. Programming of urban parks and community events;
3. Implementation of participatory planning processes.

Incorporating community-based decision-making into urban planning is essential [164]. By involving residents in the planning and design of public spaces, cities can ensure that these spaces reflect the needs and desires of the community. Participatory planning processes can strengthen social bonds and foster a sense of ownership and responsibility for public spaces, encouraging residents to actively engage with and care for their environment.

As climate change continues to exacerbate extreme weather conditions, including heatwaves, urban policies must focus on creating climate-resilient cities. Planning for resilience involves not only mitigating the effects of UHIs but also ensuring that cities are designed to support social cohesion, even in the face of environmental challenges. This may involve integrating green infrastructure with public transportation systems to encourage sustainable mobility, creating climate-adaptive public spaces that remain usable year-round, and investing in renewable energy solutions that reduce urban heat production. This approach recognizes that public transport is a crucial factor influencing social isolation and social interactions, and should be considered alongside urban greenery in comprehensive planning strategies.

In addition, urban greening policies can be incorporated into climate adaptation plans by requiring new developments to include green roofs, walls, or public green spaces as part of their design [165]. Green infrastructure retrofits for existing urban areas are particularly important, as they can address heat islands in established neighborhoods where UHI effects may be most severe. These retrofits might include adding street trees, converting vacant lots to pocket parks, installing green roofs on public buildings, and implementing rain gardens that provide both cooling effects and stormwater management benefits.

## 10. Future Research Directions

This narrative review identified several knowledge gaps that highlight the need for further research and informed the proposed future directions. One key gap is the limited understanding of the long-term effects of urban microclimate interventions, such as green spaces and shaded areas, on social isolation, as most studies focus on short-term impacts or specific case studies [166]. Additionally, while the potential for smart city technologies, such as sensors and IoT systems, to monitor urban microclimates is increasingly recognized, there is little empirical research exploring their application in tracking social behaviors or evaluating the effectiveness of interventions. Specific examples of promising technologies include temperature and humidity sensors, pedestrian counters, activity trackers, and integrated urban dashboards that can provide real-time data on both environmental conditions and human behavior patterns.

For example, while urban greening and cooling measures are widely discussed [167,168], the role of real-time data in optimizing their placement and functionality remains underexplored. The proposed future directions, such as longitudinal studies, smart city technology applications, and cross-cultural comparisons, directly address these gaps by emphasizing the need for evidence-based tools and strategies to guide urban planning. The recommendation to utilize smart city sensors is tied to these gaps, offering a method to monitor localized climate variations and assess their social implications, bridging the disconnect between emerging technologies and actionable urban interventions.

**Figure 3:**
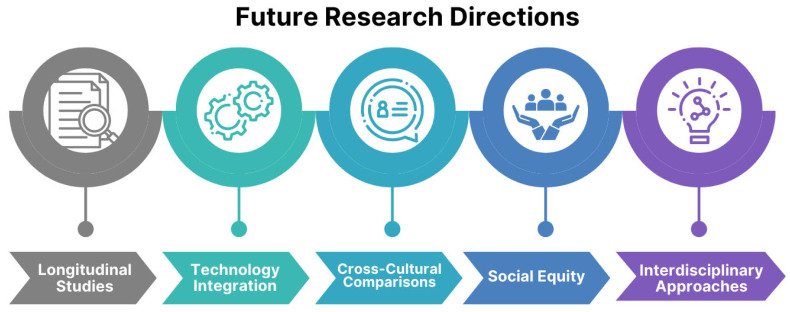
Future research directions in urban microclimates and social isolation.

Although research has increasingly highlighted the relationship between urban microclimates and social isolation [97], further investigation is required to fully understand and quantify the effects of localized climate variations on social behavior. As cities continue to face challenges related to climate change, rapid urbanization, and social fragmentation [169], it is essential to develop a deeper understanding of how environmental factors influence social engagement and well-being. Several key areas for future research can help fill these knowledge gaps and inform more effective urban planning and policy interventions. Figure 3, below, shows five key research directions: longitudinal studies, technology integration, cross-cultural comparisons, social equity, and interdisciplinary approaches.

### 10.1. Longitudinal Studies on Microclimate Interventions and Social Isolation

One critical area for future research is the need for longitudinal studies that track changes in social isolation and social behavior over time in response to urban microclimate interventions. Such studies would provide valuable insights into the long-term effects of urban greening, shaded area development, and cooling infrastructure on social cohesion. By following individuals and communities over extended periods, researchers can assess whether interventions, such as the installation of green roofs or the expansion of public parks, result in measurable improvements in social interaction, mental health, and community resilience. These studies would also help identify whether certain interventions are more effective for specific populations, such as the elderly, children, or individuals with chronic health conditions.

Moreover, longitudinal research can explore how seasonal changes and shifts in climate patterns influence the success of these interventions. For example, tracking how well green spaces mitigate social isolation during heatwaves or cold snaps would provide valuable data on the resilience of these strategies across different environmental conditions. This long-term perspective is critical to determining which interventions are most sustainable and effective in promoting social interaction, reducing isolation, and improving overall urban livability.

It is important to note that as beneficial interventions like shaded areas and urban green spaces are implemented on a large scale, finding control conditions or populations for comparative studies may become challenging. This raises ethical considerations regarding the selection of control populations who would be deprived of the benefits of these interventions. Future research designs will need to address these methodological and ethical challenges.

### 10.2. The Role of Technology in Monitoring Microclimates and Social Engagement

Advances in smart city technology offer new opportunities for monitoring urban microclimates and their impact on social behavior [170]. Future research should investigate how technologies such as smart sensors, Internet of Things (IoT) devices, and urban data platforms can be used to measure real-time variations in temperature, humidity, air quality, and other environmental factors across different urban settings. Examples of such technologies include fixed sensor networks that can monitor temperature gradients across neighborhoods, wearable sensors that track both environmental conditions and physiological responses, and computer vision systems that can analyze patterns of social interaction in public spaces.

These sensors can provide precise data on microclimatic conditions in specific locations, such as parks, streets, or residential areas, allowing researchers to correlate these conditions with patterns of social engagement or avoidance. For example, by analyzing foot traffic data, the frequency of public space usage, or the presence of individuals in social hubs, researchers can better understand how people respond to varying microclimatic conditions. This could involve studying whether residents are more likely to use shaded walkways, urban parks, or cooling centers during heatwaves, and how their behavior changes when these spaces are unavailable or inaccessible. Additionally, smart city platforms could be used to monitor the effectiveness of urban interventions, such as tree-planting programs, and determine their impact on social behavior and community cohesion [171].

Another promising area of research is the potential for virtual reality (VR) environments and augmented reality (AR) tools to simulate urban microclimates and study their effects on social behavior in controlled experiments. VR simulations could model different urban designs, climate conditions, and greening interventions, allowing researchers to observe how individuals react to these environments in terms of social interaction, comfort, and perceived isolation.

### 10.3. Cross-Cultural Comparisons of Urban Design and Social Isolation

Urban microclimates are influenced by a variety of factors, including geographic location, cultural practices, and regional climate patterns. Future research should focus on cross-cultural comparisons to assess how different urban designs, climates, and social structures affect social isolation in diverse settings. This would involve examining cities in various climates—ranging from hot, arid regions to temperate and cold environments—to determine how microclimates influence social engagement across cultures.

For example, researchers could study how cities in hot climates, such as Dubai or Mexico City, manage the effects of UHIs and whether shaded areas or cooling interventions are more effective in these regions compared to cities in temperate zones like Berlin or Copenhagen. Similarly, comparing urban greening strategies in different cultural contexts, such as dense Asian megacities versus European cities with more expansive public spaces, could provide valuable insights into how social behaviors are shaped by both environmental conditions and cultural norms.

These cross-cultural comparisons would help identify best practices that can be adapted to various urban contexts and climates. It is worth noting that urban design approaches that are effective in temperate climates might need significant adaptation for hot or cold climates. For instance, the function and design of green spaces might differ substantially between regions based on local climate conditions and cultural preferences for outdoor activities.

By understanding how different regions approach urban design, policymakers can learn from successful models of UHI mitigation and social engagement that are tailored to specific environmental and cultural conditions.

### 10.4. Social Equity and Access to Climate-Resilient Spaces

Another important area for future research is the investigation of social equity in access to climate-resilient public spaces. Vulnerable populations, such as low-income individuals, racial minorities, and those with disabilities, are often disproportionately affected by UHIs and have less access to shaded areas and green spaces [172,173]. Research should focus on understanding how inequalities in urban design contribute to disparities in social isolation and well-being. This includes exploring how different socioeconomic groups experience urban microclimates and whether policies aimed at mitigating UHIs are equitably distributed across cities.

Future studies could examine the effectiveness of urban greening programs in reducing social isolation among marginalized communities and identify barriers to accessing these spaces. Additionally, research should investigate how community-based approaches, such as participatory urban planning and citizen engagement, can improve the inclusivity and accessibility of green spaces. By focusing on social equity, researchers can help ensure that climate-resilient interventions benefit all residents, particularly those who are most vulnerable to the effects of UHIs and social isolation.

### 10.5. Interdisciplinary Approaches to Urban Microclimates and Social Behavior

Given the complex interplay between environmental conditions, social behavior, and health, future research should adopt an interdisciplinary approach that integrates urban planning, public health, sociology, and environmental science. Collaborative studies involving experts from these fields can provide a more comprehensive understanding of how urban microclimates impact social isolation and community well-being.

For example, research teams could combine qualitative data on residents’ experiences of isolation with quantitative data on environmental conditions to develop holistic models of how microclimates influence social behavior. In addition, interdisciplinary research could explore how urban policies related to housing, transportation, and infrastructure intersect with microclimate conditions to shape social behavior. By bridging the gap between different academic disciplines, future studies can inform more holistic and effective urban planning strategies that promote social resilience and environmental sustainability.

## 11. Limitations of the Review

While this review provides valuable insights into the relationship between urban microclimates and social isolation, there are several limitations that should be acknowledged. One notable limitation is the potential for geographical biases in the available research. Most studies on UHIs, green spaces, and their impact on social behavior tend to focus on urban environments in high-income countries, particularly in North America and Europe. This geographical focus may limit the generalizability of findings to other regions, especially low- and middle-income countries with different urban designs, climatic conditions, and social dynamics. As a result, the conclusions drawn from this review may not fully capture the diverse experiences of urban populations in varying climates and socioeconomic contexts.

Additionally, the review methodology has certain limitations. While we aimed for a comprehensive approach, the narrative nature of the review means that not all relevant literature may have been captured. The inclusion and exclusion criteria, while systematically applied, may have led to the omission of some valuable studies. Furthermore, the interdisciplinary nature of the topic means that relevant research might be published in journals or disciplines not fully covered by our search strategy.

The lack of longitudinal studies tracking the long-term effects of urban microclimate interventions also presents a limitation, as most studies focus on short-term impacts. Future research should address these gaps to provide a more comprehensive and globally applicable understanding of how microclimatic factors influence social isolation.

## 12. Conclusions

Urban microclimates, particularly the contrasting effects of UHIs and shaded areas, play a pivotal role in shaping social behaviors and influencing the levels of social isolation experienced by urban residents. UHIs, characterized by elevated temperatures and a lack of cooling infrastructure, tend to discourage outdoor activities, contributing to reduced social engagement and exacerbating feelings of isolation, particularly among vulnerable populations such as the elderly, children, and those with chronic health conditions. In contrast, shaded areas and green spaces provide cooler, more inviting environments that encourage physical activity, social interaction, and community bonding, serving as vital buffers against loneliness.

As urbanization intensifies and climate change amplifies the frequency and severity of extreme weather events, the need to address the social impacts of urban microclimates becomes more urgent. Urban planners and policymakers must recognize the significant influence of microclimatic conditions on social behavior and prioritize interventions that create socially inclusive and climate-resilient environments. This includes increasing vegetation, creating shaded walkways, enhancing access to green spaces, and utilizing smart city technologies to monitor and adapt to changing environmental conditions.

This review contributes to the growing body of literature linking environmental conditions to social well-being by specifically focusing on the causal pathways between urban microclimates and social isolation. The evidence presented highlights the importance of considering both physical and social dimensions in urban planning, particularly as cities worldwide face increasing challenges from climate change.

By integrating microclimate considerations into urban design and policy, cities can promote greater social cohesion, reduce the harmful effects of social isolation, and foster healthier, more resilient communities. As the global urban population continues to grow, addressing the interplay between microclimates and social interaction will be essential for creating connected, livable cities that support the well-being of all residents.

## 13. Recommendations

To effectively address the challenges posed by urban microclimates and reduce social isolation, urban planners should consider several key interventions. Firstly, the integration of technological solutions can greatly enhance the monitoring and management of microclimates. For example, smart city sensors can track localized temperature, humidity, and air quality in real-time, providing data to optimize green space placement and shading infrastructure. These sensors could also inform urban planning decisions by identifying heat-prone areas where interventions such as tree planting or reflective surfaces are most needed. Additionally, using IoT technologies can help automate climate-responsive systems, such as cooling stations and irrigation for green spaces, to ensure urban areas remain comfortable and socially engaging year-round.

Urban planners should also adhere to optimal planning guidelines for green spaces, which include ensuring that every neighborhood has equitable access to parks and shaded areas within walking distance. Research suggests that green spaces should cover at least 9 square meters per capita in urban areas to provide sufficient cooling and encourage social interaction. Designing these spaces with a focus on inclusivity—by incorporating shaded seating, accessible pathways, and areas for community events—can further foster social connections.

Moreover, planners should integrate green roofs, urban forests, and shaded walkways to reduce the UHI effect and create comfortable public spaces that promote social cohesion. These interventions should be designed for all climates, not just higher temperatures, ensuring year-round comfort and usability.

Finally, collaborative urban planning that involves local communities in decision-making processes is recommended to ensure the interventions meet residents’ needs, fostering a stronger sense of ownership and engagement with the spaces created. Urban green spaces can serve as sites for advocacy, demonstrating the importance of sustainable urban development and climate resilience. By showcasing the social, health, and environmental benefits of well-designed green spaces, researchers and planners can advocate for policies that prioritize these interventions in urban development strategies.

## Figures and Tables

**Table 1:** The relationship between urban microclimates and social behavior.

Aspect	UHIs	Shaded Areas and Green Spaces	Seasonal Variations	Time of Day
Outdoor Temperature and Social Interaction	Elevated temperatures due to heat-absorbing materials like asphalt and concrete: - Reduces likelihood of outdoor activities, limiting social encounters [60]. - Vulnerable populations (elderly, children) are disproportionately affected, leading to greater social isolation. - Public spaces (parks, markets) see decreased use during hot weather [61].	Cooler environments through tree cover, vegetation, or man-made shading systems: - Encourages outdoor activities and social behavior. - Creates inviting, comfortable spaces for longer outdoor engagement. - Supports mental well-being and fosters community interaction [62]. - Study locations include Melbourne, Australia; Copenhagen, Denmark; and urban parks in Taiwan [63,64].	- UHIs may make outdoor spaces undesirable in hot summers but beneficial in cold winters [65]. - Warmer microclimates during winter can attract outdoor activity. - Seasonal heat can limit summer social engagement while boosting winter gatherings. - Studies in London found reduced winter mortality in urban areas compared to rural areas due to the UHI effect [66].	- Outdoor activity in UHIs is often restricted to early morning or late evening during hot weather. - Cooler microclimates in shaded areas make social engagement possible throughout the day, even in hotter climates. - In colder months, UHIs retain warmth in the evenings, allowing for outdoor gatherings [67]. - Studies in Los Angeles and Phoenix showed distinct patterns of park usage concentrated in early morning and evening hours during summer months [68].
Impact on Public Space Usage	- Fewer people frequent public spaces such as parks, squares, and markets during hot weather [61]. - Reduced foot traffic and lower likelihood of spontaneous social encounters. - Heatwaves can further exacerbate social isolation by discouraging any outdoor engagement. - Melbourne research showed up to 30% reduction in public space usage during heat events [69].	- Increases use of public spaces. - Shaded parks, walkways, and green areas are popular hubs for social interaction [70]. - Supports planned and spontaneous social gatherings. - Studies from Singapore and Hong Kong demonstrated higher occupancy rates in shaded versus unshaded areas of the same parks [71].	- Warm microclimates encourage outdoor activity in winter. - Cold seasons lead to more use of warmer urban environments like plazas, markets [72]. - Summer heat reduces social interactions outdoors in UHI-prone areas. - Research in Toronto showed increased winter usage of public spaces with UHI effects compared to suburban areas [73].	- Public spaces in UHIs are more usable in cooler periods (morning, evening). - Shaded areas and green spaces are usable throughout the day, promoting year-round social interaction [74]. - Studies in Madrid found that the distribution of social activities in public spaces varied significantly by time of day during summer, concentrating in shaded areas as sun position changed [75].
Effects on Mental and Physical Health	- Prolonged indoor stays due to excessive heat contribute to mental health issues like stress and loneliness [76]. - Higher risk of physical conditions like heat exhaustion, particularly among vulnerable groups. - Increased social isolation exacerbates health problems. - Studies in New York City linked heat waves to increased emergency room visits for mental health issues [77].	- Positive effects on mental well-being due to more outdoor physical activity and social interaction. - Cooler areas reduce stress and enhance a sense of belonging and connectedness [78]. - Research in Barcelona found that regular access to urban green spaces was associated with a 15% reduction in depression indicators [79].	- Reduced stress in milder winter temperatures in UHIs. - Improved physical activity during moderate temperatures in colder months, boosting mental and physical health. - Studies in Stockholm found seasonal variation in the relationship between green space exposure and mental health outcomes [80].	- Morning and evening activities support physical health and social connections in UHI areas [81]. - Shaded areas ensure consistent mental health benefits throughout the day. - Research in Brisbane showed that time-of-day affects the perceived restorativeness of green spaces, with early morning visits associated with greater stress reduction [82].
Role in Reducing Social Isolation	- Reduces opportunities for social interaction, especially during extreme heat. - Contributes to loneliness and disconnection from the community. - Studies in Atlanta found that residents in high-UHI neighborhoods reported 20% fewer social interactions during summer months [83].	- Facilitates social connectedness through comfortable outdoor environments. - Enhances opportunities for community bonding and interaction. - Promotes social resilience by providing inclusive, accessible spaces for all. - Research in Malmö, Sweden found that neighborhoods with more green space had stronger measures of social cohesion and reduced isolation among residents [84].	- UHIs in winter mitigate cold weather, encouraging social interaction. - In hot seasons, the heat leads to decreased outdoor socializing, deepening isolation. - Studies in Chicago found that winter UHI effects reduced social isolation among elderly residents compared to suburban counterparts [85].	- Cooler periods of the day in UHIs can reduce social isolation if leveraged. - Shaded areas can help combat isolation by fostering interactions throughout the day, regardless of heat [86]. - Research in Phoenix found that community programs scheduled during cooler times of day increased participation rates among vulnerable populations by up to 35% [87].

Abbreviation: UHIs—urban heat islands.

**Table 2:** Impacts of UHI on various vulnerable populations.

Vulnerable Population	Impact of UHIs	Benefits of Shaded Areas and Green Spaces	Examples from Current Evidence
Elderly	- Heat Sensitivity: More susceptible to heat-related illnesses (heatstroke, dehydration, cardiovascular strain) due to reduced ability to regulate body temperature. - Reduced Mobility: Discomfort from heat prevents outdoor physical activities like walking, reducing exercise crucial for health and social interaction. - Social Isolation: Limited participation in community events, contributing to loneliness and mental health issues like depression and anxiety. - Health Risks: Low-income elderly often lack access to cooling systems (e.g., air conditioning), exacerbating health risks during heatwaves.	- Comfortable Outdoor Spaces: Shaded areas enable older adults to engage in physical activities like walking, which is essential for maintaining mobility and cardiovascular health. - Social Hubs: Green spaces foster social gatherings, helping elderly individuals build connections and reduce isolation. - Mental Health Benefits: Exposure to nature and social interaction in parks reduces stress, anxiety, and promotes a sense of belonging.	- Studies in Taiwan show that green spaces improve life satisfaction among older adults by promoting physical and social activity [117]. - In Macau, shaded parks have been linked to lower levels of reported loneliness among elderly residents [118]. - Research in Spain found that elderly residents living in neighborhoods with high tree canopy coverage reported 30% more social interactions during summer months than those in areas lacking shade [119].
Children	- Heat Vulnerability: Children have less capacity to regulate body temperature, making them more susceptible to heat stress during outdoor play in UHI-affected areas. - Limited Physical Activity: Excessive heat in urban areas reduces the time children can spend outdoors, limiting their physical development and social interaction. - Social Isolation: Inability to participate in outdoor group activities (e.g., sports, playgrounds) leads to fewer opportunities to build friendships and develop social skills. - Disadvantaged Children: Low-income children are disproportionately affected due to a lack of green spaces and shaded playgrounds in their neighborhoods.	- Safe Play Spaces: Shaded playgrounds and green parks provide children with safer environments for physical activities, even during hot weather. - Cognitive and Social Development: Access to parks encourages peer interaction, helping children build friendships and improve social and cognitive skills. - Mental Well-Being: Nature exposure in green spaces improves attention, creativity, and reduces behavioral issues in children.	- A study in North America and Europe found that children with regular access to green spaces had better emotional and behavioral well-being [120]. - Research from Lithuania has shown that every additional hour of time spent in parks was associated with decreased sedentary behavior and a lower risk of poor health in children [121]. - Studies in Australia found that schoolyards with increased shade provision saw a 45% increase in active play during hot weather compared to unshaded schoolyards [122].
Individuals with Chronic Health Conditions	- Exacerbation of Illnesses: Extreme heat in UHI areas worsens chronic conditions like heart disease, diabetes, and respiratory disorders, limiting individuals’ mobility and ability to participate in outdoor activities. - Increased Sedentary Behavior: Heat prevents individuals from engaging in outdoor exercise, exacerbating conditions like obesity and cardiovascular disease. - Social Withdrawal: Chronic health issues combined with heat-related discomfort increase the likelihood of staying indoors, which amplifies feelings of isolation and disconnection from the community.	- Health Supportive Environments: Green spaces provide cooler environments where individuals with chronic conditions can safely engage in light physical activity, such as walking, without exacerbating their health conditions. - Stress Reduction: Access to nature reduces stress, lowers blood pressure, and improves mental health, which is especially important for people with chronic illnesses. - Community Engagement: Shaded areas in parks offer opportunities for individuals to participate in group activities (e.g., tai chi, yoga), fostering social connections and reducing isolation.	- Research in Canada found that people with respiratory issues were significantly more likely to use green spaces due to the cooling and air-purifying benefits of vegetation and there was a decline of mortality because of this [109]. - Public Health England review in 2020 revealed that many of the greatest health challenges in society today have changed since 19th century health campaigners first saw the benefit of securing open spaces to act as the lungs of our cities, creating purer air and giving people places for healthful exercise and social engagement [123]. - Studies in Berlin found that patients with cardiovascular conditions who had access to residential green space engaged in 25% more outdoor physical activity than those without such access [124].
Low-Income Individuals	- Limited Access to Cooling: Low-income individuals often live in areas with less vegetation and fewer green spaces, meaning they experience higher temperatures and are more exposed to the negative effects of UHIs. - Economic Barriers: Lack of resources for air conditioning or cooling systems leaves low-income individuals more vulnerable to heat stress and heat-related illnesses. - Health Disparities: The compounding effects of poor access to healthcare, limited mobility, and the inability to escape the heat create significant health risks. - Social Inequality: Living in UHI-prone areas with fewer public spaces reduces opportunities for social engagement, reinforcing social and health disparities.	- Promotes Social Equity: Public green spaces in low-income neighborhoods provide accessible environments for relaxation, exercise, and socialization, helping reduce disparities in health and well-being. - Cooling Solutions: Shaded areas provide free, accessible cooling relief for low-income individuals who cannot afford air conditioning. - Community Building: Green spaces act as inclusive gathering points for community events, fostering social cohesion and reducing the isolation felt in economically marginalized areas.	- Studies in Los Angeles found that low-income neighborhoods with more tree cover experienced fewer heat-related illnesses during extreme heat events [125]. - Research in Melbourne shows that urban greening projects in low-income areas resulted in improved social connectedness and reduced feelings of exclusion among residents [126]. - Community-led greening initiatives in Philadelphia’s low-income neighborhoods led to a 28% increase in reported neighborly interactions and a measurable decrease in crime rates [127].
People with Disabilities	- Heat Intolerance: Certain disabilities, especially those affecting mobility, make individuals more susceptible to heat-related health problems as they may struggle to move to cooler areas. - Restricted Mobility: Heat can make navigating public spaces more difficult for individuals with physical disabilities, leading to further isolation. - Barrier to Participation: Excessive heat limits the participation of people with disabilities in outdoor social and recreational activities, deepening feelings of loneliness.	- Accessibility: Shaded areas with well-designed, accessible infrastructure enable people with disabilities to participate in outdoor activities and social interactions. - Inclusive Spaces: Green spaces that are inclusive and equipped with accessibility features can promote engagement in community life, reducing the isolation experienced by individuals with disabilities. - Mental Health Benefits: Nature-based settings help alleviate stress and improve mental health for individuals with disabilities, who often face barriers to social participation.	- Research in Copenhagen indicates that accessible green spaces reduce mobility barriers for people with disabilities, promoting outdoor activity and social inclusion [128]. - Studies from Berlin highlight the importance of green spaces in improving the mental well-being of individuals with disabilities, especially during summer months [129]. - Research in Toronto found that accessible shaded areas in parks increased visitation rates among people with disabilities by 40% during summer months [130].

Abbreviation: UHIs—urban heat islands.
